# TNF-α-Induced VEGF and MMP-9 Expression Promotes Hemorrhagic Transformation in Pituitary Adenomas

**DOI:** 10.3390/ijms12064165

**Published:** 2011-06-23

**Authors:** Zhengzheng Xiao, Qin Liu, Feng Mao, Jun Wu, Ting Lei

**Affiliations:** 1 Department of Neurosurgery, Tongji Hospital, Tongji Medical College, Huazhong University of Science and Technology, Wuhan 430030, China; E-Mails: xzzxzz@yahoo.cn (Z.Z.X.); maofeng.tongji@yahoo.cn (F.M.); zma@nccu.edu (J.W.); 2 Department of Pediatrics, Union Hospital, Tongji Medical College, Huazhong University of Science and Technology, Wuhan 430030, China; E-Mail: qliuunion@yahoo.cn

**Keywords:** pituitary apoplexy, intratumoral hemorrhage, TNF-α, VEGF, MMP-9

## Abstract

Pituitary apoplexy is a clinical syndrome with unknown pathogenesis. Therefore, identifying the underlying mechanisms is of high clinical relevance. Tumor necrosis factor alpha (TNF-α) is a critical cytokine mediating various hemorrhagic events, but little is known about its involvement in pituitary apoplexy. Here we show that TNF-α may be an important regulator of hemorrhagic transformation in pituitary adenomas. In this study, sixty surgical specimens of hemorrhagic and non-hemorrhagic human pituitary adenomas were examined. Hemorrhagic pituitary adenomas displayed higher protein and mRNA levels of TNF-α, vascular endothelial growth factor (VEGF) and matrix metalloproteinase-9 (MMP-9) compared with those of non-hemorrhagic tumors. Exposure of MMQ pituitary adenoma cells to TNF-α induced VEGF and MMP-9 expression *in vitro*. Additionally, TNF-α administration caused hemorrhagic transformation and enhanced VEGF and MMP-9 expression in MMQ pituitary adenoma cell xenografts in mice. Blockers of VEGF or MMP-9, either alone or in combination, attenuated but not abrogated TNF-α mediated hemorrhagic transformation in xenografts. This study suggests that TNF-α may play a role in the development of intratumoral hemorrhage in pituitary adenomas via up-regulation of VEGF and MMP-9.

## 1. Introduction

Pituitary apoplexy is a potentially life-threatening syndrome resulting from hemorrhage, hemorrhagic infarction or infarction of a pre-existing pituitary adenoma [1]. The reported incidence of apoplectic pituitary tumors varies from 0.6 to 10% of all surgically resected adenomas [2]. Pathological studies show that more than 60% of apoplectic pituitary adenomas display hemorrhage or hemorrhagic infarction [3]. However, the mechanisms underlying hemorrhagic transformation in pituitary adenomas remain unclear. Increasing evidence from recent studies suggests that TNF-α plays a critical role in the development of various hemorrhagic events [4–6]. TNF-α is a well-known cytokine involved in multiple pathological processes such as angiogenesis, destruction of vascular integrity, and vascular hyperpermeability [7], which promote hemorrhagic transformation [6]. The underlying mechanism has been proposed to be TNF-α mediated up-regulation of VEGF and MMP-9 [6,8–11].

VEGF, a growth factor which has essential roles in vasculogenesis, angiogenesis and vascular permeability [12], has been correlated with a propensity for hemorrhage in various tumors and vascular diseases. VEGF overexpression in U87 MG cells and U251 MG cells causes intratumoral hemorrhage in their xenografts [13,14]. VEGF administration aggravates hemorrhagic transformation in rats after transient focal cerebral ischemia [15]. The involvement of VEGF in the development of pituitary adenoma hemorrhage remains controversial. Arita *et al*. [16] found a positive relation between hemorrhage and VEGF protein expression in 39 human pituitary adenomas. However, other studies found no significant relation [17–19].

MMP-9 is known to degrade extracellular matrix around blood vessels leading to increased vascular permeability [20,21]. MMP-9 has been associated with hemorrhagic events in many clinical entities, such as aneurysmal diseases [6], intraventricular hemorrhage in premature infants [22], metastatic brain tumors [8], anovulatory dysfunctional uterine bleeding [23], and arteriovenous malformations [9]. Moreover, hemorrhagic pituitary adenomas exhibit higher MMP-9 protein expression levels than non-hemorrhagic tumors [24]. As suggested by previous findings, up-regulation of either VEGF or MMP-9 can cause instability of vessels [25], thereby predisposing to hemorrhagic events.

Based on the fact that TNF-α and TNFR1 are present in normal and tumoral anterior pituitary cells [26,27], we hypothesized that TNF-α may enhance MMP-9 and VEGF expression, which, in turn, contributes to hemorrhagic transformation in pituitary adenomas via degrading components of vascular basement lamina and increasing vascular permeability. In the present study, the possible correlation between TNF-α and hemorrhage within pituitary adenomas was investigated. We evaluated TNF-α, VEGF and MMP-9 expression in surgical specimens of hemorrhagic and non-hemorrhagic pituitary adenomas. Furthermore, the association between TNF-α and hemorrhagic presentation was also identified *in vitro* and *in vivo*.

## 2. Results and Discussion

### 2.1. Clinical Features of Patients

In our study, there were 30 patients harboring hemorrhagic adenomas (6 men, 24 women; age range 17 to 62 years, mean age 40.50 ± 13.00 years) and 30 patients with non-hemorrhagic adenomas (8 men, 22 women; age range 22 to 55 years, mean age 37.50 ± 9.94 years). The numbers of clinical and subclinical pituitary apoplexy patients were 8 and 22, respectively. The eight patients with clinical pituitary apoplexy had several common clinical symptoms, including headache (8/8), nausea (7/8), vomiting (7/8), visual field defect (7/8), decreased visual acuity (6/8), ophthalmoplegia (4/8), febrile (3/8), and altered consciousness (1/8). For the patients (*n* = 30) with hemorrhagic pituitary adenomas, histopathological assessment revealed that 18 patients had prolactinomas, five patients had non-functional adenomas, three had multihormonal adenomas, two had gonadotropinomas and two had GH-secreting adenomas. In the patients (*n* = 30) with non-hemorrhagic pituitary adenomas, 21 patients had prolactinomas, five patients had non-functioning adenomas, two had gonadotropinomas, and two had GH-secreting adenomas. Invasive tumors were found in four patients (hemorrhagic tumor in one patient and non-hemorrhagic tumors in three). Intratumoral hemorrhage was not observed to correlate with tumor size, hormone types, angiogenesis and tumor invasion (data not shown).

### 2.2. Increased Expression of TNF-α, VEGF and MMP-9 in Hemorrhagic Pituitary Adenoma Tissues

**Figure 1:**
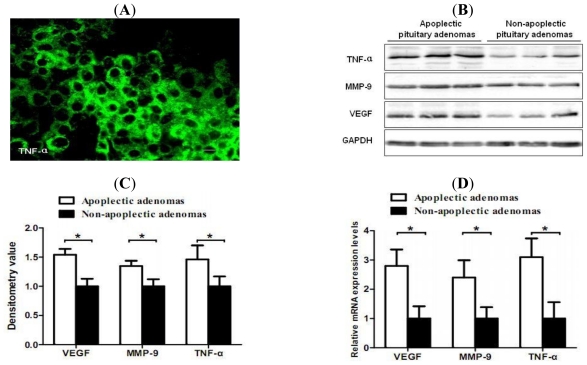
Increased expression levels of tumor necrosis factor alpha (TNF-α), vascular endothelial growth factor (VEGF) and matrix metalloproteinase-9 (MMP-9) in hemorrhagic pituitary adenomas. (**A**) Representative immunofluorescence staining of TNF-α. Scale bar = 5 μm; (**B**) Western blot results showed higher expression levels of TNF-α, VEGF and MMP-9 in hemorrhagic pituitary adenomas (*P <* 0.05); (**C**) Densitometric analysis normalized with GAPDH as loading control was also shown; (**D**) Real-time RT-PCR was performed with the same tissues and showed increased mRNA expression levels of TNF-α, VEGF and MMP-9 (*P <* 0.05). All of the bars in the figures show mean ± SD. * *P* < 0.05.

To determine whether TNF-α is associated with intratumoral hemorrhage in pituitary adenomas, we examined TNF-α expression in hemorrhagic and non-hemorrhagic pituitary adenomas. Immunofluorescence demonstrated prominent positive cytoplasmic specific staining of TNF-α in pituitary adenomas (Figure 1A). Western blotting showed increased protein levels of TNF-α in hemorrhagic pituitary adenomas compared with non-hemorrhagic tumors (*P* < 0.05). To further elucidate the potential mechanisms by which TNF-α induces hemorrhage in pituitary adenomas, the expression levels of VEGF and MMP-9 were determined. Western blotting showed that the protein levels of TNF-α, VEGF and MMP-9 in hemorrhagic pituitary adenomas were significantly higher than those in non-hemorrhagic specimens (*P* < 0.05) (Figure 1B). The mRNA levels of VEGF and MMP-9 were significantly higher in hemorrhagic pituitary adenomas (*P* < 0.05) (Figure 1C).

### 2.3. Correlation of TNF-α and Hemorrhage-Associated Genes Expression in Hemorrhagic and Non-Hemorrhagic Pituitary Adenoma Tissues

As statistically significant higher expression levels of TNF-α protein were found in hemorrhagic pituitary adenomas, correlations between TNF-α and hemorrhage-associated genes (VEGF and MMP-9) expression in pituitary adenoma tissues were analyzed. The Pearson correlation coefficients for VEGF and MMP-9 versus TNF-α protein expression in pituitary adenomas were 0.674 (*P* = 0.01) and 0.521 (*P* = 0.01), respectively.

### 2.4. Hemorrhagic Incite Has no Effect on MMP-9 and VEGF Expression

**Figure 2:**
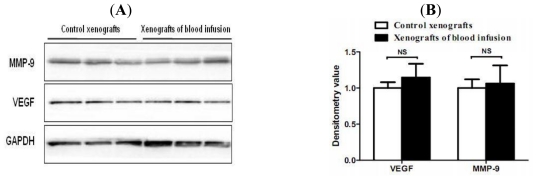
Effect of blood injection on protein expression levels of VEGF and MMP-9 in xenograft tumors. Whole blood (500 μL) or 0.9% saline was injected into subcutaneous xenograft tumors (3 tumors per condition), after two days, tumors were harvested and protein was extracted for western blot. (**A**) Representative western blots of xenografts from the blood injection group and the control group. No significant enhancing effect of blood injection on protein expression of VEGF and MMP-9 was observed; (**B**) Densitometric analysis normalized with GAPDH as loading control was also presented. Data are expressed as mean ± SEM. NS: not significant.

Although hemorrhagic pituitary adenomas display higher levels of VEGF and MMP-9 than those in non-hemorrhagic tumors, this phenomenon may be an effect rather than a cause of hemorrhagic incite. To clarify this question, we injected whole blood (500 μL) or 0.9% saline into subcutaneous MMQ pituitary tumor cell xenografts and then analyzed the effect of blood injection on protein expression of VEGF and MMP-9 in these tumors. The results indicated that blood has no significant enhancing effect on protein expression of VEGF and MMP-9 in MMQ cell xenografts (Figure 2).

### 2.5. TNF-α Inhibits Proliferation and Increases Hemorrhage-Associated Genes Expression of MMQ Cells *in Vitro*

**Figure 3:**
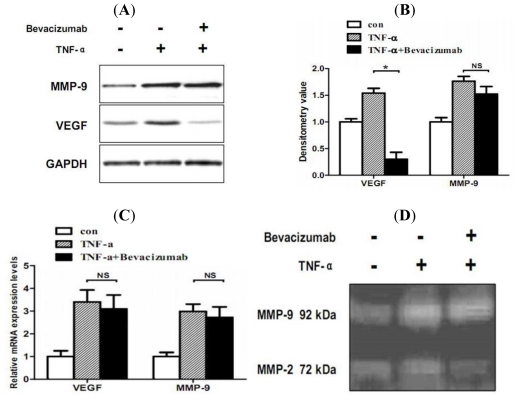
Effect of TNF-α on VEGF and MMP-9 expression in lactotroph MMQ rat pituitary tumor cells. Cells were treated with TNF-α (20 ng/mL) or pretreated with bevacizumab (5 mg/mL) for 72 h and an additional 24 h with TNF-α (20 ng/mL), and then VEGF and MMP-9 expression were determined via western blot analysis (**A**, **B**) and real-time RT-PCR (**C**). The supernatant of cells was also subjected to zymography (**D**). TNF-α induced higher protein and mRNA levels of VEGF and MMP-9 expression. Bevacizumab attenuated TNF-α mediated VEGF expression but had no effect on TNF-α mediated MMP-9 expression. Ratios of density were individually normalized with GAPDH. The bars show mean ± SEM (*n* = 3). *, *P* < 0.05. NS: not significant.

To define the role of TNF-α in stimulating hemorrhage-associated genes expression, the effect of TNF-α on cell proliferation and hemorrhage-associated genes (VEGF and MMP-9) expression was examined in the MMQ pituitary adenoma cell line. MMQ cells were treated with different concentrations of TNF-α (0, 10, 20 ng/mL) for 24 h. A dose-dependent inhibitory effect on cell proliferation was observed. After a 24 h incubation period, TNF-α stimulation at concentrations of 10 and 20 ng/mL inhibited basal cell growth by 9 ± 3.4% (*P* < 0.05) and 16 ± 4.2% (*P* < 0.05), respectively. Based on this result, we used TNF-α at concentration of 20 ng/mL for following stimulation. After 24 h of TNF-α (20 ng/mL) exposure, MMQ cells showed significantly higher protein and mRNA levels of VEGF and MMP-9 expression (*P* < 0.05), as determined by western blot analysis (Figure 3A,B) and real-time RT-PCR (Figure 3C). To determine whether VFGF regulate MMP-9 bioavailability in MMQ cells, the effect of VEGF inhibitor bevacizumab on TNF-α mediated VEGF and MMP-9 expression were investigated. Bevacizumab significantly inhibited TNF-α mediated VEGF up-regulation, while blocking of VEGF did not affect TNF-α mediated MMP-9 up-regulation. This trend was also confirmed in zymography (Figure 3D), the zymographic densitometry value for the TNF-α group was 1.15 ± 0.12, and 1.21 ± 0.19 for the bevacizumab plus TNF-α group (*P* > 0.05).

### 2.6. TNF-α Causes Hemorrhagic Transformation and Increases Hemorrhage-Associated Genes Expression in MMQ Cell Xenografts in Mice

**Figure 4:**
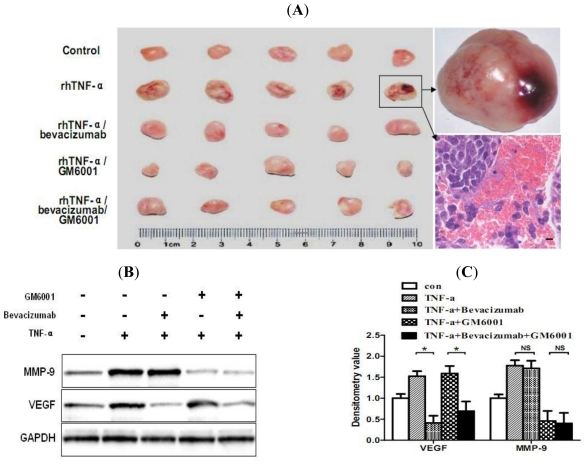
Mouse tumor hemorrhage, pathology and the expression levels of VEGF and MMP-9. Mice were implanted with MMQ cells. After two weeks, mice were treated with saline, rhTNF-α, rhTNF-α plus bevacizumab, rhTNF-α plus GM6001, rhTNF-α plus bevacizumab and GM6001. Five mice in each group. rhTNF-α (10,000 U/kg i.m.), bevacizumab (10 mg/kg i.p.) and GM6001 (10 mg/kg i.p.) were implemented twice per week. Both bevacizumab and GM6001 were administrated one week before and during TNF-α treatment. Tumors were harvested after one week rhTNF-α treatment. (**A**) Tumors of each group and representative pictures of hemorrhagic tumor and HE staining. Scale bar = 5 μm; (**B**) Protein expression levels of VEGF and MMP-9 in tumors after treatment. Western blot analysis revealed the increased VEGF expression in tumors of rhTNF-α and rhTNF-α/GM6001 treated mice and increased MMP-9 expression in those of rhTNF-α and rhTNF-α/bevacizumab treated mice. Bevacizumab inhibited VEGF expression with reduced appearance of VEGF band. GM6001 inhibited MMP-9 expression with reduced appearance of MMP-9 band. (**C**) Densitometric analysis of western blot results. GAPDH was an internal control to normalize gel loading. * *P* < 0.05. NS: not significant.

To translate our above *in vitro* findings into an *in vivo* model of TNF-α-dependent hemorrhagic transformation, next we evaluated the effect of rhTNF-α on MMQ cell tumor xenograft growth in athymic nude mice. HE staining showed that rhTNF-α administration of one week caused marked hemorrhage in xenograft tumors (5 out of 6) in comparison to vehicle control (0 out of 6) (Figure 4A). We next investigated the possible mechanisms underlying rhTNF-α mediated hemorrhagic transformation *in vivo*. Western blot analysis clearly showed increased protein levels of MMP-9 and VEGF in the xenograft tumors treated with rhTNF-α compared with the control (Figure 4B). As MMP-9 appeared not to be affected by VEGF in the *in vitro* study, we tested whether suppression of VEGF or MMP-9, either alone or in combination, could reduce TNF-α mediated hemorrhagic transformation in nude mice. Using a VEGF inhibitor (bevacizumab) and a broad MMP inhibitor (GM6001), we administered either or both of them to different groups of mice one week before and during TNF-α treatment, and then the rate of TNF-α induced hemorrhage in each group was examined using HE staining. One out of five mice in the VEGF plus MMP-9 inhibitors group displayed hemorrhagic xenograft tumors, while two in the VEGF inhibitor group and three in the MMP-9 inhibitor group were found (Figure 4A). The results indicate that VEGF or MMP-9 inhibitors, either alone or in combination, could attenuate TNF-α mediated hemorrhagic transformation in MMQ cell xenograft tumors. Together, these results clearly show that TNF-α may contribute to the development of hemorrhage in MMQ tumor xenografts via upregulation of VEGF and MMP-9.

### 2.7. Discussion

The mechanism underlying hemorrhagic transformation in pituitary adenomas remains unclear. In the present study, we provide the first evidence that TNF-α plays a key role in hemorrhagic transformation in pituitary adenomas. Our results reveal an association of TNF-α elevation with up-regulation of VEGF and MMP-9 in hemorrhagic pituitary adenomas and demonstrate that TNF-α promotes hemorrhagic transformation in MMQ pituitary adenoma cell xenografts via up-regulation of VEGF and MMP-9.

TNF-α is a critical inflammatory cytokine that plays a central role in initiating and promoting inflammatory responses [4,6]. Previous studies have demonstrated that TNF-α participates in many hemorrhagic pathologic processes. TNF-α promoter polymorphisms have been found to be associated with hemorrhage in brain arteriovenous malformation, spontaneous deep intracerebral hemorrhage, and hemorrhage in trauma [5,9,28]. Recently TNF-α has been reported to be involved in aneurysm formation, growth and rupture [4]. In addition to aforementioned, TNF-α is present in both normal and tumor pituitary cells, indicating a possible role for TNF-α in the development of pituitary adenomas. In this study, elevated TNF-α level was associated with hemorrhagic pituitary adenomas, and TNF-α administration promoted hemorrhagic transformation in MMQ pituitary adenoma cell xenografts. These findings indicate an important role of TNF-α in hemorrhagic transformation of pituitary adenomas.

Considering that TNF-α may be involved in hemorrhagic transformation of pituitary adenomas, we next explored the possible mechanisms underlying its role in hemorrhagic transformation. TNF-α has been shown to stimulate VEGF expression [10]. VEGF induces not only angiogenesis and vascular hyperpermeability, but also microvessel vasodilation [12]. All these changes may predispose vessels to rupture, leading to various hemorrhagic events. In addition to experimental evidence aforementioned [13–15], elevated VEGF levels have been demonstrated in hemorrhagic pleural effusion of lung cancer [29], vitreous hemorrhage [30], cerebral hemorrhagic infarction and hemorrhage of retinal angiomas [31,32]. In terms of pituitary adenoma, studies dealing with the association between VEGF expression and pituitary adenoma hemorrhage have been controversial [16–19]. The discrepancy may be due to the differences in patient selection, sample sizes and hormone-secreting types of tumors studied. Another explanation is that the development of intratumoral hemorrhage may require different threshold levels of VEGF in different patients, because the absolute levels of VEGF are critical to its function in both physiological and pathological conditions [13]. This explanation is exemplified in part by the 4-fold elevation of VEGF secretion required for an acute development of hemorrhage in glioma cell xenografts [13].

In addition to VEGF, MMP-9 is also known to be regulated by TNF-α and has been implicated in hemorrhagic transformation [11]. MMP-9 has broad substrate specificity and degrades several extracellular matrix components. MMP-9 activation may lead to destruction of microvascular integrity and vascular remodeling, which results in hemorrhagic events [20,21]. Hemorrhagic pituitary adenomas express higher levels of MMP-9 [24], suggesting a possible role for MMP-9 in the development of hemorrhage within pituitary adenomas. In this study, the TNF-α mediated up-regulation of VEGF and MMP-9 was confirmed in MMQ cells. These data are consistent with previous findings of TNF-α in other human cells [6,10,33]. Moreover, the inefficiency of VEGF inhibitor in preventing TNF-α mediated MMP-9 expression exclude the possible direct effects of VEGF on MMP-9 expression in MMQ cells.

In this study, elevated VEGF and MMP-9 expression in hemorrhagic pituitary adenomas was demonstrated. Furthermore, TNF-α administration induced VEGF and MMP-9 expression and caused hemorrhagic transformation in MMQ cell xenografts. These findings confirm the pivotal role of TNF-α in mediating VEGF and MMP-9 up-regulation in hemorrhagic transformation of pituitary adenomas. Contrary to our expectations, the application of both VEGF and MMP-9 inhibitors attenuated but not abrogated TNF-α mediated hemorrhagic transformation. These results suggest that, besides VEGF and MMP-9, other targets may also contribute to hemorrhagic transformation. Our *in vivo* results may raise a question as whether the remaining hemorrhagic transformation in tumors treated with inhibitors is caused by the inhibitor drug itself. However, in this study, inhibitor treatment for one week before the TNF-α administration had not caused obvious intratumoral hemorrhage in xenograft tumors during the week. This observation excluded the possibility of side effect of the inhibitory drugs on hemorrhage transformation.

There is an inherent limitation of our experimental results from patient samples, because whether they are a cause or an effect of hemorrhagic incites remains unclear. However, the effects of hemorrhage have been reported to generally abate over the first week in animal studies [34,35]. As our institution is a tertiary care medical center, there was a considerable delay between the symptom onset and referral to our clinic in our patients. The median duration of symptom prior to surgery was two weeks in our patients. In addition, we also investigated samples from subclinical pituitary adenoma patients who might have even longer duration of hemorrhage prior to surgery [36]. We found there was no significant difference between clinical and subclinical hemorrhagic pituitary adenoma tissues in the western blot results. These two kinds of hemorrhagic tissues both displayed higher protein levels of TNF-α, VEGF and MMP-9 than non-hemorrhagic tumors. Furthermore, we also determined the effect of blood injection on MMP-9 and VEGF protein expression in xenograft tumors and found no significant difference between tumors of blood group and those from control group. This result also suggests that elevated MMP-9 and VEGF protein levels may be a cause rather than an effect of hemorrhagic incite in pituitary adenomas.

Apoplectic pituitary adenomas display three different pathologic types: hemorrhage, hemorrhagic infarction and infarction, and these three pathologic types are present in nearly equal numbers [3]. Moreover, about 30% of patients presenting with pituitary tumor apoplexy have precipitating factors or associated events [2]. Thus, it seems likely that multiple mechanisms may be involved in the pathogenesis of pituitary adenoma apoplexy. This study confirmed that TNF-α promoted hemorrhagic transformation via up-regulation of VEGF and MMP-9 in a mice model of pituitary tumor cell xenografts. The data support the view that TNF-α mediated up-regulation of VEGF and MMP-9 is a contributory cause for hemorrhagic transformation in pituitary adenomas. Although TNF-α cannot explain all the pathology seen in pituitary adenoma hemorrhage, it is clearly an important component to the pathology. TNF-α may act alone or perhaps in concert with environmental exposures, risk factors or other diseases to promote the development of pituitary adenoma hemorrhage. Besides TNF-α, there may be many other mechanisms underlying pituitary adenoma hemorrhage, which warrants further studies.

## 3. Materials and Methods

### 3.1. Patients and Samples

The study was approved by the Research Ethics Committee of the Huazhong University of Science and Technology, Wuhan, China. Prior informed consent was obtained from all patients enrolled in this study. All specimens were made anonymous according to ethical standards.

From May 2009 to December 2010, a series of 60 surgically removed pituitary adenomas were obtained from patients undergoing transsphenoidal surgery at the Department of Neurosurgery, Tongji Hospital, Huazhong University of Science and Technology, Wuhan, China. Each sample was bisected. One half was immediately frozen at −80 °C until protein and RNA extraction. The other half was fixed with 10% formalin and embedded in paraffin for histology and immunofluorescence.

### 3.2. Histology and Immunofluorescence

The sections were deparaffinized and rehydrated in successive xylene and alcohol, and then stained with Hematoxylin and Eosin for the morphological diagnosis. For immunofluorescence, paraffin sections of tumors were deparaffinized and rehydrated by successive washes with xylene, graded ethanol. Antigen retrieval was accomplished in microwave oven in 100 mM sodium citrate buffer (pH 6.0) for 10 min and then cooled to room temperature for 20 min. Subsequently, slides were incubated with 10% normal serum followed by the primary anti-TNF-α antibody (Millipore, 1:500) and incubated overnight at 4 °C. The slides were then incubated with corresponding Alexa fluor-labeled secondary antibody (Invitrogen) for 30 min. For quantification, 3 to 5 “hotspot” areas were captured for each slide by two individuals. Digital images were analyzed and quantified using Image-Pro Plus.

### 3.3. Cell Culture

MMQ, a rat prolactin-secreting pituitary tumor cell line, was purchased from ATCC (American Type Culture Collection, Manassas, VA) and routinely cultured in F-12 medium supplemented with 2.5% fetal bovine serum and 15% equine serum at 37 °C in a humidified air atmosphere containing 5% carbon dioxide in T-75 flasks. MMQ cells were stimulated with 20 ng/mL TNF-α (Abcam) or saline for 24 h, and then were analyzed for possible effects of TNF-α on VEGF and MMP-9 expression. In order to evaluate the impact of VEGF blockade on TNF-α mediated MMP-9 expression in MMQ cells, another group of cells were pretreated with VEGF inhibitor bevacizumab (Roche) (5 mg/mL) for 72 h and then TNF-α (20 ng/mL) was added for an additional 24 h. After incubation, cells were harvested for western blot and real-time RT-PCR analysis.

### 3.4. Cell Viability Assay

The effect of TNF-α on cell viability was carried out using Cell Counting Kit-8 (Boster Co.). Briefly, MMQ cells were plated in 96-well plates (20,000/well). The cells were treated with TNF-α at concentrations of 0, 10, 20 ng/mL. After 24 h incubation, 10 μL of the CCK-8 solution was then added to each well of the plate, and then the plate was incubated for another 1 h. The absorbance was measured at 450 nm using a 96-well plate reader.

### 3.5. Western Blot Analysis

Expression of VEGF and MMP-9 protein from adenoma tissues, cells and xenograft tumors were determined by western blot analysis. Tumors or cells were thawed on ice and homogenized at 4 °C in lysis buffer for extracting total protein. The protein was quantitated using a bicinchoninic acid (BCA) reagent. Equivalent amounts of protein (30 μg) from each sample were separated, under denaturing conditions, by electrophoresis on 10% SDS-PAGE gels (polyacrylamide gels containing sodium dodecyl sulfate). The protein bands were transferred by electroblotting to nitrocellulose membranes. The blots were incubated 1 hour with PBS contain 0.1% Tween 20 (PBST) and 5% powdered milk, and then incubated overnight with anti-human/rat MMP-9 antibody (Santa Cruz, 1:1000), anti-human/rat VEGF antibody (Santa Cruz, 1:1000) and anti-human/rat GAPDH antibody (Cell Signaling, 1:3000). The membranes were washed six times with TBST (10 mM Tris·HCl, pH 8.0/150 mM NaCl/0.1% Tween 20), and then were incubated with relative second antibodies and washed six times with TBST. Bands were visualized by ECL (Thermo-Pierce). In each tumor sample, protein levels were normalized to that of GAPDH as a loading control.

### 3.6. Zymography

MMQ cells (1 × 10^6^) were cultured with TNF-α (20 ng/mL) or TNF-α plus VEGF inhibitor bevacizumab (5 mg/mL) in serum free DMEM medium, after 24 h incubation, the supernatant was collected, and centrifuged at 2000 rpm for 10 min. Protein concentration was determined and electrophoresed on 7.5% non-reducing SDS–PAGE gels containing 2 mg/mL gelatin as a substrate at constant voltage. After electrophoresis, the gels were rinsed in renaturation buffer (2.5% Triton X-100) on a shaker for 30 min to remove SDS and were incubated overnight at 37 °C in a water bath in activation buffer composed of 50 mmol/L Tris-HCl (pH 7.5), 5 mmol/L CaCl_2_. Gels were stained using 0.5% Coomassie blue R-250 (Bio-Rad) for 2 h, followed by appropriate destaining. MMP activity was detected as a band on a dark blue background and quantified densitometrically using Image-Pro Plus.

### 3.7. Real-Time RT-PCR

Total RNA was isolated from cell culture and frozen tumor tissues using RNeasy Mini kit (QIAGEN) according to the manufacturer’s instructions. Real-time RT-PCR was done using TaqMan one-step RT-PCR master mix kit and gene specific taqman gene expression assay kits in a 7300 real-time PCR system (Applied Biosystems). PCR amplification was carried out in a 20 μL reaction volume containing 200 ng of cDNA, 1.8 μM each of forward and reverse primers, and 10 μL Fast SYBR Green Master Mix (Applied Biosystems). The PCR reactions were initiated with denaturation at 95 °C for 10 min; followed by 40 amplification cycles at 95 °C for 15 s and 60 °C for 1 min. Samples were run in triplicate and results were averaged. CT was calculated by analysis software SDS 2.2.1 (Applied Biosystems). Analysis of gene expression changes were calculated using the 2-ΔΔCT method. The normalized expression ratio (fold induction) was calculated by 2-ΔΔCT = fold induction. Statistical analyses were performed using the CT ± SD values. The primer sequences used for real-time RT-PCR for Human TNF-α F: TGGAGAAGGGTGACCGACTC, R: TGCCCAGACTCGGCAAAG; Human MMP-9 F: CCCTGGAGACCTGAGAACCA, R: CCCGAGTGTAACCATAGCGG; Human VEGF F: TGTGAATGCAGACCAAAGAAAG, R: GCTTTCTCCGCTCTGAGCAA; Human GAPDH F: GAAGGTGAAGGTCGGAGTC R: GAAGATGGTGATGGGATTTC; Rat VEGF F: TGTGCGGGCTGCTGCAATGAT, R: TGTGCTGGCTTTGGTGAGGTTTGA; Rat MMP-9 F: CTTTGTAGGGTCGGTTCTG, R: GAGTGGATAGCTCGGTGG; Rat GAPDH F: GTCTTCACTACCATGGAGAAGG, R:TCATGGATGACCTTGGCCAG; Real-time PCR results were carried out by three replicate measurements of each sample.

### 3.8. Animal Xenografts

Six week-old athymic nude mice were inoculated subcutaneously in the lower rear flank with MMQ cells suspended in 0.1 mL of PBS (1 × 10^6^ cells per mouse inoculation). rhTNF-α, bevacizumab and GM6001 were purchased from Weike Shanghai Biopharmaceutical Limited, Roche and Chemicon, respectively. Mice were randomized into five groups: control (saline) group, rhTNF-α group, rhTNF-α plus bevacizumab (a VEGF inhibitor) group, rhTNF-α plus GM6001 (a broad MMP inhibitor) group, rhTNF-α plus bevacizumab and GM6001 group, with five mice in each group. Treatments were started two weeks after cell injection. rhTNF-α (10,000 U/kg i.m.), bevacizumab (10 mg/kg i.p.) and GM6001 (10 mg/kg i.p.) were implemented twice per week. Both bevacizumab and GM6001 were administrated one week before and during rhTNF-α treatment. Tumors were harvested after one week of rhTNF-α treatment. Half of each tumor was frozen in liquid nitrogen for protein analysis and the other half was fixed in 10% formalin for histology analysis.

### 3.9. Blood Infusion in Xenograft Tumors

To investigate whether hemorrhagic incite affects MMP-9 and VEGF expression in pituitary adenoma tissues, we injected whole blood (500 μL) or 0.9% saline into subcutaneous MMQ cell xenografts, respectively. Two days after injection, the mice were sacrificed and tumors were collected. Tumors from whole blood group (3 cases) and saline group (3 cases) were immediately frozen and then western blot analysis was performed for protein expression of MMP-9 and VEGF.

### 3.10. Statistical Analysis

Statistical analyses were processed and analyzed with the aid of SPSS 17.0 software. For experiment with patient samples, results are expressed as the mean ± SD. For *in vitro* and *in vivo* studies, values were expressed as mean ± SEM. Student *t*-tests were used to compare hemorrhagic adenomas *versus* non-hemorrhagic tumors on protein and mRNA levels. Pearson correlation test was used to determine the correlations between VEGF, MMP-9 and TNF-α protein expression in tissues. Results from animal and cell culture studies were analyzed using Student *t*-tests. Probability values of less than 0.05 were considered statistically significant.

## 4. Conclusions

Taken together, our results show that TNF-α significantly correlates with intratumoral hemorrhage in pituitary adenomas and induces pituitary adenoma hemorrhage through up-regulation of VEGF and MMP-9. Based on these findings, our study demonstrates that TNF-α may play a role in the development of hemorrhage within pituitary adenomas.
